# Complications in multiple gestation pregnancy: A cross-sectional study of ten maternal-fetal medicine centers in China

**DOI:** 10.18632/oncotarget.9000

**Published:** 2016-04-26

**Authors:** Jun Wei, Qi-Jun Wu, Tie-Ning Zhang, Zi-Qi Shen, Hao Liu, Dong-Ming Zheng, Hong Cui, Collaborative Group on Twin Birth, Fetal Abnormality in China, Cai-Xia Liu

**Affiliations:** ^1^ Department of Obstetrics and Gynecology, Shengjing Hospital of China Medical University, Shenyang, China; ^2^ Department of Clinical Epidemiology, Shengjing Hospital of China Medical University, Shenyang, China; ^3^ Department of Pediatrics, Shengjing Hospital of China Medical University, Shenyang, China

**Keywords:** China, cross-sectional study, multiple births, pregnancy complications

## Abstract

Complications in women with multiple gestation pregnancy have not been studied in China. We aimed to establish a database of women with multiple gestation pregnancy and investigate the complications related to multiple pregnancy. We conducted a cross-sectional study that included 3246 women with multiple gestation pregnancy and who had multiple live-birth deliveries; the women were registered at ten maternal-fetal medicine centers in China in 2013. All participants completed a detailed questionnaire that included basic demographic information, history of gestation and abnormal fetal development, risk factors during pregnancy, and pregnancy outcomes. Overall, 1553 (47.8%) women experienced pregnancy complications; these women were more likely to have lower height and less education than women who did not experience complications. However, women who experienced complications had a higher twin birth rate and were more likely to have received regular antenatal care and assisted reproductive technology than women without complications (*P* < 0.05). Notably, preterm birth was a primary complication in multiple pregnancy (*n* = 960). In conclusion, pregnancy complications, especially preterm birth, were relatively common in women with multiple gestation pregnancy. The findings from this cross-sectional study in China may be used as a foundation for investigating risk factors for complications in women with multiple gestation pregnancy in the future.

## INTRODUCTION

Multiple gestation pregnancies result from complex interactions among genetic and environmental factors. Hereditary influences, older maternal age, assisted reproductive technology (ART), and advanced parity are well-established risk factors for multiple pregnancy [1]. Multiple pregnancy and multiple live-birth rates have increased during recent decades and multiple births now account for 3% of all births worldwide [2–4]. The rate of twin birth increased by more than 75% in the United States from 1980 to 2009 [5]. Similar trends have been observed in Western Europe and other countries [4].

Nevertheless, multiple pregnancy is considered a high risk for obstetric complications such as spontaneous abortion, hypertensive disorders, placenta previa, and fetal malformations [6]. Specifically, the incidence of hypertensive disorders, a common source of maternal morbidity, is 15% to 35% in twin pregnancies, which is two to five times higher than in singleton pregnancies [7–12]. Additionally, the etiology of preterm birth is not completely understood, but the association between multiple pregnancy and preterm birth is well known [13]. A secondary analysis of the WHO Global Survey dataset indicated that 35.2% of multiple births were preterm (< 37 weeks gestation); of all multiple births, 6.1% of births were before 32 weeks gestation, 5.8% were during weeks 32 and 33, and 23.2% were during weeks 34 through 37 [14]. Multiple-birth infants also have a substantially higher risk of perinatal mortality and, globally, multiple births account for 14% of all infant deaths [5].

The incidence of multiple birth in China is rising [15], but no recent studies have examined pregnancy complications in women with multiple pregnancy using a multi-center database. The two-child policy was established in China in 2016, so a baby-boom period is expected in the next decade and, with it, an increase in multiple gestation pregnancies. We conducted this cross-sectional study at ten hospitals in China to summarize complications in women with multiple pregnancy. We hope our findings will contribute to the establishment of diagnostic standards and treatments for complications of multiple pregnancy.

## RESULTS

We included a total of 3246 women with multiple gestation pregnancy in our analysis. The majority of women delivered twins (*n* = 3101, 95.5%). Table 1 presents the numbers of women with multiple pregnancy and pregnancy complications at each of the ten hospitals included in our database. Of these hospitals, the Women's Hospital School of Medicine at Zhejiang University had the most participants (*n* = 538, 16.6%). The Shandong Provincial Hospital of Shandong University had the lowest proportion of twin births (*n* = 275, 82.3%). The proportion of pregnancy complications varied among the ten hospitals; the Women's Hospital School of Medicine at Zhejiang University had the highest proportion of women with multiple pregnancy who experienced complications (*n* = 457, 84.9%) and the Shanghai First Maternity and Infant Health Institute Affiliated Tongji University had the lowest proportion (*n* = 25, 6.1%). The average proportion of pregnancy complications was 47.8% (*n* = 1553) for all ten hospitals. Table 2 lists the numbers and types of pregnancy complications at each of the ten hospitals.

**Table 1:** The numbers of women with multiple gestation pregnancy and pregnancy complications at each of the ten hospitals

Hospitals	No. Multiples births (%)	No. Twin births (%)*	No. pregnancy complications (%)^†^
First Affiliated Hospital of Chongqing Medical University	180 (5.5)	177 (98.3)	71 (39.4)
First Affiliated Hospital of Sun Yat-sen University	206 (6.3)	200 (97.1)	131 (63.6)
Gulou Clinical Medical College of Nanjing Medical University	159 (4.9)	157 (98.7)	81 (50.9)
Obstetrical and Gynecological Hospital affiliated Fudan University	468 (14.4)	461 (98.5)	180 (38.5)
Peking University First Hospital	156 (4.8)	149 (95.5)	77(49.4)
Shandong Provincial Hospital to Shandong University	334 (10.0)	275 (82.3)	131 (39.2)
Shanghai First Maternity and Infant Health Institute Affiliated Tongji University	409 (12.6)	408 (99.8)	25 (6.1)
Shengjing Hospital of China Medical University	430 (13.2)	393 (91.4)	221 (51.4)
Third Hospital affiliated GuangZhou Medical University	366 (11.3)	353 (96.4)	179 (48.9)
Women's Hospital School of Medicine Zhejiang University	538 (16.6)	528 (98.1)	457 (84.9)
Overall	3246	3101 (95.5)	1553 (47.8)

* The proportions of twin births were calculated as the proportion of all multiples births at each hospital.

† The proportions of pregnancy complications were calculated as the proportion of all multiples births at each hospital. Pregnancy complications included gestational hypertension, gestational diabetes mellitus, preterm birth, and pregnancy complicated with internal medicine, surgical, or infectious disease.

**Table 2:** The numbers of pregnancy complications at each of the ten hospitals

Hospitals	No. Gestational hypertension (%)	No. Gestational DM (%)	No. Preterm birth (%)	No. Pregnancy complicated with internal medicine, surgical, and infectious disease (%)
First Affiliated Hospital of Chongqing Medical University	16 (4.7)	30 (7.1)	26 (2.7)	10 (3.0)
First Affiliated Hospital of Sun Yat-sen University	27 (7.9)	35 (8.3)	70 (7.3)	54 (16.2)
Gulou Clinical Medical College of Nanjing Medical University	4 (1.2)	23 (5.5)	54 (5.6)	11 (3.3)
Obstetrical and Gynecological Hospital affiliated Fudan University	65 (19.1)	60 (14.3)	66 (6.9)	23 (6.9)
Peking University First Hospital	10 (2.9)	28 (6.7)	49 (5.1)	7 (2.1)
Shandong Provincial Hospital to Shandong University	55 (16.1)	13 (3.1)	70 (7.3)	34 (10.2)
Shanghai First Maternity and Infant Health Institute Affiliated Tongji University	11 (3.2)	18 (4.3)	0 (0)	0 (0)
Shengjing Hospital of China Medical University	48 (14.1)	65 (15.4)	124 (12.9)	28 (8.4)
Third Hospital affiliated GuangZhou Medical University	18 (5.3)	55 (13.0)	90 (9.4)	61 (18.3)
Women's Hospital School of Medicine Zhejiang University	87 (25.5)	94 (22.3)	411 (42.8)	106 (31.7)
Overall	341	421	960	334

Table 3 presents the distribution of characteristics of women with multiple pregnancy with and without pregnancy complications. Women with complications had significantly lower height (*P* = 0.05) and education level (*P* < 0.01) than women without complications; however, women with complications had a higher rate of twin birth (*P* = 0.05) and were more likely to have received regular antenatal care (*P* < 0.05) and ART (*P* < 0.05).

**Table 3:** Characteristics of women with multiple pregnancy with and without pregnancy complications

Characteristic	Total	Pregnancy with complications*	Pregnancy without complications	*P*-value
Number of women	3246	1553	1693	
**Mean (SD)**				
Age (years)	30.2 (7.0)	30.2 (4.5)	30.0 (4.3)	0.17
Weight (kg)	55.6 (8.9)	55.5 (8.7)	55.5 (8.2)	0.35
Height (cm)	161.2 (5.4)	160.7 (4.9)	161.3 (5.0)	0.05
**No. (%)**				
Twin births	3101 (95.5)	1498 (96.5)	1603 (94.7)	0.05
**Education level**				
Above bachelor degree	232 (7.1)	96 (6.0)	136 (8.0)	< 0.01
Alcohol drinking (yes)	4 (0.1)	2 (0.2)	2 (0.09)	0.89
Cigarette smoking (yes)	4 (0.1)	3 (0.3)	1 (0.04)	0.61
Regular antenatal care	2129 (65.6)	1213 (78.1)	916 (54.1)	< 0.05
ART	1574 (48.5)	788 (50.7)	786 (46.4)	< 0.05
IVF-ET	1260 (80.1)	656 (42.2)	604 (35.7)	
Induction of ovulation	196 (12.5)	87 (5.6)	109 (6.4)	
Artificial insemination and others	118 (7.5)	45 (5.7)	73 (9.3)	

ART, assisted reproductive technology; IVF-ET, *in vitro* fertilization and embryo transfer; SD, standard deviation.

* Pregnancy complications included gestational hypertension, gestational diabetes mellitus, preterm birth, and pregnancy complicated with internal medicine, surgical, or infectious disease.

Preterm birth was one of the primary complications of multiple pregnancy in our analysis (Table 4). In all, 960 women delivered preterm infants. Additionally, 341 women were diagnosed with gestational hypertension and 421 with gestational diabetes mellitus. Also, 334 women had pregnancies complicated by internal medicine, surgical, or infectious disease. These complications have significantly different characteristics from each other (data not shown).

**Table 4:** Characteristics of women with multiple pregnancy according to complications

Characteristic	Gestational hypertension	Gestational DM	Preterm birth	Pregnancy complicated with internal medicine, surgical, or infectious disease
No. of cases	341	421	960	334
**Mean (SD)**				
Age (years)	31.7 (4.6)	31.6 (4.0)	29.9 (4.5)	30.3 (4.7)
Weight (kg)	56.4 (8.0)	57.2 (9.7)	54.9 (8.5)	55.5 (8.3)
Height (cm)	161.2 (5.4)	160.7 (4.9)	160.9 (5.0)	160.9 (5.1)
**No. (%)**				
Twin births	326 (95.6)	410 (97.4)	925 (96.4)	320 (95.8)
**Education level**				
Above bachelor degree	241 (70.7)	238 (56.5)	51 (5.3)	219 (65.6)
Alcohol drinking (yes)	2 (0.6)	2 (0.5)	0	0
Cigarette smoking (yes)	4 (1.2)	3 (0.7)	0	0
Regular antenatal care	235 (68.9)	346 (82.2)	801 (83.4)	261 (78.1)
ART	164 (48.1)	243 (57.7)	467 (48.6)	173 (51.8)
IVF-ET	140 (85.4)	212 (87.2)	389 (83.3)	143 (82.7)
Induction of ovulation	21 (12.8)	20 (8.2)	57 (12.2)	21 (12.1)
Artificial insemination and others	3 (1.8)	5 (2.1)	21 (4.5)	6 (3.5)

ART, assisted reproductive technology; DM, diabetes mellitus; IVF-ET, *in vitro* fertilization and embryo transfer; SD, standard deviation.

## DISCUSSION

Evidence of pregnancy complications in women with multiple gestation pregnancy is limited in China. To our knowledge, this is the first multi-center, cross-sectional study in China to describe complications in women with multiple pregnancy. Our findings indicate that women with pregnancy complications have lower height and education level but higher rates of twin birth, regular antenatal care, and ART than women without complications. Preterm birth was the most common pregnancy complication. The database from ten maternal-fetal centers could be used to investigate risk factors for complications in women with multiple pregnancy in the future.

In our analysis, 341 (9.95%) women were diagnosed with gestational hypertension. This is consistent with the study by Ghulmiyyah et al. [16], which found that 8% to 10% of pregnant women worldwide experience gestational hypertension. Additionally, 421 (12.97%) women were diagnosed with gestational diabetes. Hollander et al. [17] concluded that 2% to 10% of pregnancies, including multiple and singleton pregnancies, are affected by diabetes mellitus. The exact reason for the difference between Hollander's findings and ours is not clear, but it might be attributed to differences in populations, diets, and research methods. Further studies are warranted to confirm the rates of diabetes in pregnancy. In our study, 960 (29.57%) women delivered preterm infants. The incidence of preterm delivery has been reported to be approximately 10% [18]. The higher rate observed in our study might result simply from the fact that we included only multiple pregnancies, which are frequently associated with preterm birth.

Little evidence of the effects of ART on maternal and fetal well-being is available [19]. Several meta-analyses and reviews have proposed that ART singleton pregnancies are associated with higher risks of adverse obstetric outcomes [20–23]. In our study, we found that the rate of pregnancy complications in women who received ART was significantly higher than in women who did not receive ART (*P* < 0.01). This finding was consistent with previous reports [24]. Specifically, Daniel et al. [25] found that women who received ART had higher incidences of pregnancy-induced hypertension, uterine bleeding, and fetal death than women who did not receive ART. We observed a possible link between ART and pregnancy complications, but further study is needed to confirm the association in women with multiple pregnancies and help obstetricians manage the risks associated with multiple pregnancy.

There are several strengths of this study. To our knowledge, this is the first cross-sectional study to assess the incidence of pregnancy complications in women with multiple pregnancy in China. We collected data from the ten largest maternal-fetal medicine centers in China in order to establish a national database for pregnancy complications in women with multiple pregnancy. All of the pregnancy complications were diagnosed by physicians and recorded through the hospital information systems. Since the information was not self-reported, we are confident in reporting results and drawing conclusions from the data. However, several limitations of our study should be considered. First, the study was a cross-sectional design. Unlike prospective studies, cross-sectional studies cannot examine etiologic objectives or confirm hypotheses. Additionally, cross-sectional studies often pose difficulty in determining the timing of events and they are associated with length-biased sampling. All of the hospitals included in our analysis were regional maternal-fetal medicine centers, which made it difficult to conduct long follow-up of the women with multiple pregnancy after parturition. Further, although we collected data from the ten largest maternal-fetal medicine centers in China, Berkson's bias might appear in the study, which could overestimate the rates of pregnancy complications in twin births. Our findings should be interpreted with caution in pregnant women living in rural or less-developed areas.

In conclusion, the data from this cross-sectional study of women with multiple pregnancy in China may be used as baseline information not only to provide scientific evidence for the prevention and diagnosis of pregnancy complications but also to establish a population-based database of multiple pregnancies and births for health care and research purposes.

## MATERIALS AND METHODS

### Study populations

We performed a multi-center, cross-sectional study that analyzed women with multiple pregnancy in 2013. Each of the ten hospitals included in this study was a Grade 3 facility affiliated with a university. The hospitals were located in different regions of China, and they were among the first hospitals approved by the National Health and Family Planning Commission of the People's Republic of China to establish local regional maternal-fetal medicine centers. The institutional review board of each facility approved our study. The ten hospitals were: First Affiliated Hospital of Sun Yat-sen University, First Affiliated Hospital of Chongqing Medical University, Gulou Clinical Medical College of Nanjing Medical University, Obstetrical and Gynecological Hospital Affiliated to Fudan University, Peking University First Hospital, Shandong Provincial Hospital of Shandong University, Shanghai First Maternity and Infant Health Institute Affiliated to Tongji University, Shengjing Hospital of China Medical University, Third Hospital Affiliated at GuangZhou Medical University, and Women's Hospital School of Medicine at Zhejiang University.

To be eligible for the study, women must have had a multiple gestation pregnancy, including normal twins and abnormal twins. Pregnant women who were diagnosed with a twin pregnancy by ultrasound in early pregnancy were also included. Women who were pregnant with fetuses with trisomy 21, trisomy 18, or trisomy 13 were not included in this study. In all, a total of 3246 women with multiple pregnancy from ten hospitals were included in the analysis; the response rate was 95.9%.

### Exposure measurement

We extracted data from a pre-existing database that contained various demographic and delivery information, including select obstetric and neonatal outcomes for all women who delivered at the hospitals. The data were entered by nurses or physicians who attended the deliveries; quarterly audits ensured the accuracy of data entry. Each woman included in our study provided informed consent, and nurses or physicians were then allowed to collect information from the hospital information system during a woman's hospitalization. When quality or completeness of the data was questionable, an in-person interview was conducted to complete the questionnaire. The questionnaire comprised the following four sections: 1) basic demographic information, 2) history of gestation and abnormal fetal development, 3) risk factors during pregnancy, and 4) pregnancy outcomes.

For example, we classified study participants as ever smokers or drinker if they had ever smoked cigarettes or drank alcohol. For women who received assisted reproductive technology, we also collected the method they received (*in vitro* fertilization and embryo transfer, induction of ovulation, and artificial insemination and others). The information of weight and height prior to give birth were collected during a woman's hospitalization.

### Outcomes assessments

Pregnancy complications in women with multiple pregnancy included hypertensive disorders, gestational diabetes mellitus, preterm birth, pregnancy complicated with internal medicine disease, pregnancy complicated with surgical disease, and pregnancy complicated with infectious disease. The diagnostic criteria for hypertensive disorders in pregnancy included clinical diagnosis by an obstetrician during pregnancy and systolic/diastolic blood pressure greater than 140/90 mmHg with or without proteinuria [26]. The diagnostic criteria for gestational diabetes mellitus included clinical diagnosis by an obstetrician during pregnancy and a 75-g oral glucose tolerance test that resulted in a fasting blood glucose level of greater than 5.1 mmol/L, a 1-hour postprandial blood glucose level greater than 10.0 mmol/L, or a 2-hour postprandial blood glucose level greater than 8.5 mmol/L [27]. The diagnostic criteria for preterm birth was delivery between 28 and 37 weeks gestation [28]. The diagnostic criteria for pregnancy complicated with internal medicine disease included clinical diagnosis by an obstetrician during pregnancy and physician-diagnosed disease of the cardiovascular system, respiratory system, digestive system, blood system, or any other body system. The diagnostic criteria for pregnancy complicated with surgical disease included clinical diagnosis by an obstetrician during pregnancy and surgeon-diagnosed gastrointestinal disease, orthopedic disease, trauma, or other surgical disease or complication. The diagnostic criteria for pregnancy complicated with infectious disease included clinical diagnosis by an obstetrician during pregnancy and diagnosis by an infectious diseases specialist of hepatitis, syphilis, AIDS, or other infectious disease.

### Statistical analyses

We calculated the means and percentages of selected variables for pregnancies with and without complications. We compared the data using *t*-tests for continuous variables and Pearson χ^2^ tests for categorical variables. Statistical analyses were conducted using SPSS 16.0 (SPSS Institute, Chicago, Illinois, USA), and a two-sided *P*-value of 0.05 was considered statistically significant.
